# The Breath

**Published:** 1893-01

**Authors:** J. Taft


### The Breath.

BY J. TAFT, D.D.S.

Read before the Ohio State Dental Society, December 6, 1892.

It is not the intention in this paper to enter into a consideration of the subject of respiration in its physiological features, nor to raise an inquiry as to the normal changes that are accomplished in the process of respiration, but rather to give expression to a few thoughts upon some of the abnormal conditions in which the breath is so frequently found.

There is a great variety of vitiated conditions of the products from the respiratory apparatus; this is a subject of great interest and importance.

Impure breath is liable to occur at any and all periods of life. This is experienced in the variations of health condition.

It is found in a great variety of phases--from the slightest perceptible deviation from the normal state to that in which it is loaded with a large amount and great variety of excrementitious matter from various sources, perceived sometimes even by the sense of taste, at least so far as the patient is concerned, but more especially by the olfactories, making such an impression through this channel as to produce nausea, and, indeed, the infection of contagious diseases may be carried from one person to another by the breath. A vitiated breath as a means of conveying the seeds of diseases is not duly appreciated by the public, and perhaps not so fully as it ought to be by the majority of physicians. The various conditions of the breath, when rightly interpreted, in many instances, indicate much in respect to active diseases; undoubtedly this is true in regard to diphtheria, scarlatina, typhoid, and probably some other diseases; and in others of a more or less perverted or vitiated physiological action, which the physician would do well to note and study.

In the social relations of life, how often does an offensive breath stand as an obstacle to a nearness of intercourse that on every other account would be acceptable and even desirable. We involuntarily turn away from the fetid breath of even our best friends. One may be beautiful in face and form, attractive in manner, and fascinating in conversation, and possess personal magnetism that may be well nigh irresistible, and yet an offensive breath will neutralize or destroy the influence or power of all these qualities.

In no other relation is this subject of greater moment than that existing between the dentist and his patient; they are brought into such contact that each must, in a degree, at least, be brought in contact with the expired breath of the other, and this usually for more than a brief time.

The degree of offensiveness is modified by the acuteness of the olfactories of one or both parties, together with the conditions of the exhalations, and the source or sources of the fetid breath.

To the dentist this is a subject of serious import, as it is liable to affect his comfort, his health, his work, and his business. In the first three of these particulars the offense is with the breath of the patient, the last has reference to his own condition.

The dentist always suffers more or less discomfort from the offensive breath of his patient; this is especially so, if his sense of smell is very acute, and if the odor from the breath is very marked. This sometimes produces nausea, and sometimes acts depressingly upon the nervous system, these may occur even in the milder forms of fetid breath.

If the dentist is subjected for a considerable time to this condition of things his general health may become affected in a more or less marked degree, according to his own susceptibility, and to the degree of vitiation of the air he is compelled to inhale. There can hardly be a doubt but what many suffer very greatly from this cause; and though one may not suffer much, or apparently at all in this respect, yet a sense of discomfort and uneasiness is always experienced by the dentist in his professional work when subjected to a vitiated atmosphere, and this will necessarily interfere somewhat with the thoroughness of his operations. It is a serious question whether the dentist ought ever to subject himself to such embarrassments, at least, for more than a few minutes at a time. It certainly would be for the welfare and interest of both the patient and the dentist were the former to be put upon proper treatment before having extended operations made upon the teeth.

The offensive breath of the operator is a matter of special interest to the patient as well as to himself. The dentist has no right to make himself, or even to be, a nuisance in any respect to those whom he serves, and if he has any just appreciation of the fitness of things he will see to it, that his presence in every respect is rendered as little objectionable as possible.

The patient is not very likely to suffer ill health or prolonged discomfort from the fetid breath of the dentist, but the highly sensitive patient, and, indeed, many of less acute sensibility, will be inclined by and by, if not sooner, to seek more acceptable service.

The late Dr. Hawxhurst, in writing upon this subject, says: "It is folly for the practitioner to under-rate the influence which a bad breath may exert in driving his patients from him. Under my own observation several operators have suffered seriously from this cause.

The first feeling that comes into the mind on smelling a disagreeable odor is a desire to get away from it. The dentist whose breath is infected with odors that disgust must not be surprised if his patients adopt speedy means of putting themselves at a safe distance from this infection. No other single influence can equal it in repelling those on whom he is to rely for his support. The dentist should never operate unless he knows his breath is pure. To operate from one to two hours, and during the whole time a stream of fetid odor poured directly into the face of a sensitive patient, is a gross insult, and one to which very few will submit without some sort of protest."

The dentist should be able at all times to discriminate in regard to the character of his own breath; it may sometimes be a necessity to submit to this annoyance from others, but he should see to it that he never imposes upon his patients in this way. I have known superior operators—persons of gentlemanly deportment in every other respect, whose breath was in such a condition as to disgust all those who came within its influence; indeed, I have known some such who were compelled to abandon the practice of dentistry.

There are some instances in which this affection seems uncontrollable, but in the great majority of cases it is amenable to proper treatment, and can either be modified, masked or wholly eradicated. It can in many instances be wholly, and by proper care permanently, relieved. In others the change is only of a temporary character, irrespective of any treatment that may be employed.

This affection arises from two general sources, viz.: systemic and local. Each of these classes have quite a variety of causes, and if we properly study this subject, attention must be given to both these varieties. The first class, viz.: systemic, I will not attempt to discuss further than to say that any derangement of the general eliminating processes and channels of the body, or a derangement or diseased condition of any of the eliminating organs is likely to result in a vitiated condition of the breath. Elimination of the waste material of the organization is chiefly through five or six sets of organs; any one or more of these failing in the proper exercise of function is followed either by the derangement of other functions or of throwing vicarious work upon other parts. Derangement of the stomach, alimentary tract, kidneys, liver or skin is almost certain to result in more or less marked change of the breath, from the fact that in part, at least, the waste that thus fails to be removed is thrown into the lungs, and will, in many instances, produce a markedly offensive condition of the breath. But in some instances the breath may be contaminated with excrementitious matter that possesses little or no offensive odor. The defective function of the digestive apparatus is, in nearly all cases a source of fetid breath. Disease of the lungs of almost every variety is attended with more or less vitiation of the breath.

Of the local sources of this difficulty there are many, and of these there may be said to be two classes, the one embracing all the local disorders that may contaminate the breath after it leaves the lungs; this will embrace the various forms of diseases found in the throat, mouth and nose. Diphtheria, scarlet fever, tonsilitis and perhaps some other affections, though affecting the entire system, possess a local manifestation that results in greatly vitiated breath.

The various catarrhal affections that are found in the nose, the throat and mouth in all cases more or less affect the breath, varying very much, it is true, in different cases, ranging all the way from the very mild, almost imperceptible change to an intolerably disgusting degree. This affection should be well studied by the dentist, in order that he may be able to give his patients some, if not permanent relief, and that he may protect himself so far as he may against an intensely annoying and offensive condition.

Diseases of the gum and mucous membrane of the jaws are often the occasion of this offensive condition. This will result, sometimes, from a vitiated exudate from the mucous membrane, or it may occur, as is frequently the case, from a discharge from the margins of the gums, and from the sockets of the teeth.

Necrosis and sloughing of the bony tissues of the sockets nearly always produce a very offensive condition.

The discharge from alveolar abscess is oftentimes so vitiated as to load the breath which passes out of the mouth with an exceedingly offensive odor.

Decayed teeth are charged, especially by various medical writers, as a very frequent cause of offensive breath; this is true not only of physicians, but to a greater extent, perhaps, by the laity. There is not, however, as much in this as is usually attributed to it. Occasionally cases are presented in which an exceedingly foul breath is wholly attributed to one small innocent cavity of decay in the grinding surface of a molar tooth. Were all other causes of offensive breath eliminated, than that which comes directly from decay of the teeth, there would be, in the aggregate, an immense improvement.

Another fruitful source of offensive odors of the oral cavity is found in the presence of foreign substances or matter in the mouth in the shape of soft salivary calculus, accumulation of food, and a glutinated mucus deposited upon the teeth or artificial dentures undergoing decomposition, and necessarily throwing off an effluvia that will be mixed with the breath. The saliva and mucus mixed thus with foreign substance, and retained for an undue time in the mouth, will undergo such change as to present a very offensive condition. A simple test, such as placing the mixed saliva in a vessel at the temperature of the mouth for twelve to thirty-six hours, will abundantly verify this statement, and when food substances and epithelial scales are added to the mass the change becomes more marked and more offensive. Now as to these extraneous causes of the affection under consideration it is not difficult for the dentist nor for the educated patient to determine what should be done; simply purification of the oral cavity in the most thorough manner by the entire removal of all offensive material, and after this the intelligent use of disinfectants upon the teeth, mucous membrane and dental plates, if they are in the mouth.

These three sources of affection may exist in either the patient or the operator, and with both they should all be relieved so far as possible. For that arising from the third class of causes, viz: the presence of extraneous substances, there is no apology for it remaining as a source of trouble. In the management of the second class of causes, viz: those arising from local affections of the mouth, throat or nose, there is little excuse for these remaining as an offense to either the operator or the patient. It is true that some of these diseases are very persistent in their hold upon the tissues or upon the parts in which they are situated; but the intelligent and well educated dentist will be able, in a great many instances, to suggest treatment that will be of permanent value to the patient, and in some cases result in a permanent cure. But with a large variety of disinfectants, antiseptics, cleansing materials and methods there is no difficulty in rendering almost every such case free from the objectionable condition, for a time at least, sufficient for necessary operations.

A great many formulas have been given for the correction of offensive breath; the suggestions made for the use of these, however, in the majority of cases, are upon a false basis; with many of them it is only the substitution of one odor for another, or the mixing of two offensive conditions, and producing a third that is, perhaps, temporarily more tolerable than either of the others.

In treatment here, however, the aim should be, as in all other medical treatment, to attain the most permanent results; that, doubtless, is the true theory of all medical and surgical practice.

Temporizing should never be employed when something better can be attained. It is very desirable that the profession should give more attention to this subject than hitherto. In our literature very little will be found upon this subject, and in all medical literature, so far as I have been able to examine, only a fugitive reference to it has been here and there made; and I may here refer those who have not investigated the subject to a little work, entitled: "The Breath, and the Disorders which give it a Fetid Odor," by Dr. Joseph W. Howe, the third edition of which was issued in 1885, and a paper published in THE DENTAL REGISTER, by Dr. D. C. Hawxhurst, Vol. 27, page 104.

